# PGH_1_, the Precursor for the Anti-Inflammatory Prostaglandins of the 1-series, Is a Potent Activator of the Pro-Inflammatory Receptor CRTH2/DP2

**DOI:** 10.1371/journal.pone.0033329

**Published:** 2012-03-19

**Authors:** Ralf Schröder, Luzheng Xue, Viktoria Konya, Lene Martini, Nora Kampitsch, Jennifer L. Whistler, Trond Ulven, Akos Heinemann, Roy Pettipher, Evi Kostenis

**Affiliations:** 1 Molecular-, Cellular- and Pharmacobiology Section, Institute for Pharmaceutical Biology, University of Bonn, Bonn, Germany; 2 Biomedical Research Centre, NDM Experimental Medicine, University of Oxford, Oxford, United Kingdom; 3 Institute of Experimental and Clinical Pharmacology, Medical University Graz, Graz, Austria; 4 Ernest Gallo Clinic and Research Center, University of California San Francisco, Emeryville, California, United States of America; 5 Department of Physics, Chemistry and Pharmacy, University of Southern Denmark, Odense, Denmark; 6 Oxagen Ltd, Abingdon, Oxon, United Kingdom; Medical School of Hannover, United States of America

## Abstract

Prostaglandin H_1_ (PGH_1_) is the cyclo-oxygenase metabolite of dihomo-γ-linolenic acid (DGLA) and the precursor for the 1-series of prostaglandins which are often viewed as “anti-inflammatory”. Herein we present evidence that PGH_1_ is a potent activator of the pro-inflammatory PGD_2_ receptor CRTH2, an attractive therapeutic target to treat allergic diseases such as asthma and atopic dermatitis. Non-invasive, real time dynamic mass redistribution analysis of living human CRTH2 transfectants and Ca^2+^ flux studies reveal that PGH_1_ activates CRTH2 as PGH_2_, PGD_2_ or PGD_1_ do. The PGH_1_ precursor DGLA and the other PGH_1_ metabolites did not display such effect. PGH_1_ specifically internalizes CRTH2 in stable CRTH2 transfectants as assessed by antibody feeding assays. Physiological relevance of CRTH2 ligation by PGH_1_ is demonstrated in several primary human hematopoietic lineages, which endogenously express CRTH2: PGH_1_ mediates migration of and Ca^2+^ flux in Th2 lymphocytes, shape change of eosinophils, and their adhesion to human pulmonary microvascular endothelial cells under physiological flow conditions. All these effects are abrogated in the presence of the CRTH2 specific antagonist TM30089. Together, our results identify PGH_1_ as an important lipid intermediate and novel CRTH2 agonist which may trigger CRTH2 activation *in vivo* in the absence of functional prostaglandin D synthase.

## Introduction

The prostaglandin D_2_ (PGD_2_) receptor CRTH2 (chemoattractant receptor homologous molecule expressed on T helper type 2 (Th2) cells) appears to play a pivotal role in allergic diseases by influencing migration of inflammatory cells such as eosinophils, basophils and Th2 cells [1]–[8]. Pharmacological inhibition of CRTH2 is associated with a reduction in airway inflammation and decreased levels of mucus, Th2 cytokines and immunoglobulin E [9]–[15]. The central role played by CRTH2 in orchestrating inflammatory responses suggests that antagonism of this receptor might represent an attractive strategy to combat allergic diseases. A hallmark of CRTH2 is that it is not exclusively activated by PGD_2_, but responds to a rather broad spectrum of endogenous ligands. Among those are the PGD_2_ metabolites 13,14-dihydro-15-keto-PGD_2_, Δ^12^-PGD_2_, PGJ_2_, 15-deoxy-Δ^12,14^-PGJ_2_, and Δ^12^-PGJ_2_ [16]–[20], but interestingly also prostanoids generated independently of PGD synthase activity such as the thromboxane metabolite, 11-dehydro-TXB_2_ [21], and the PGF synthase-dependent, PGF_2α_ [20]. Activation of CRTH2 by prostanoids generated independently of the PGD synthase allows for the possibility of CRTH2 signaling *in vivo* in the absence of PGD_2_ production and thus reinforces the importance of this receptor in the orchestration of allergic inflammation.

PGH_1_ is generated from dihomo-γ-linolenic acid (DGLA) by the action of cyclo-oxygenases (COX) 1 and 2 and represents the precursor for the 1-series of prostaglandins which have been mainly viewed as anti-inflammatory [22]–[27]. PGH_2_, on the other hand, is generated from arachidonic acid (AA), the major long chain polyunsaturated fatty acid in mammalian cell membrane phospholipids and is a precursor for the 2-series of prostaglandins [28]–[30]; see **Figure S1** for pathways of prostaglandin production. Most 2-series prostaglandins have been tested for bioactivity on CRTH2 and a number of receptor-activating lipids have been identified [17], [3], [31]. However, potential modulation of CRTH2 by the 1-series of prostaglandins including their precursors has not yet been examined. Such investigations appear obligatory given the recent discovery that 1-series prostaglandins are likely to be formed *in vivo* upon ingestion of DGLA [32] and the widespread promotion of diets enriched with this poly-unsaturated fatty acid to ameliorate inflammatory lung diseases including asthma [33].

In this study we identify PGH_1_, the precursor for lipid mediators with anti-inflammatory potential, as potent and efficacious agonist for the pro-inflammatory receptor CRTH2. We characterize its bioactivity using the novel dynamic mass redistribution (DMR) technology (Corning® Epic® Biosensor) that permits non-invasive, label-free analysis of receptor signalling in living cells and in real time [34], [35]. We also provide evidence that CRTH2 activation by PGH_1_ is detectable in human eosinophils and Th2 cells and leads to their chemotactic activation, and migration, respectively.

## Materials and Methods

### Reagents

Tissue culture media and reagents were purchased from Invitrogen (Karlsruhe, Germany). DGLA, all prostaglandins, and HQL79 were from Cayman Chemicals (Ann Arbor, MI, USA) and TM30089 (CAY10471) was synthesized according to previously published procedures [36]. All other reagents were obtained from Sigma (Taufkirchen, Germany) unless explicitly indicated.

### Cell culture of CRTH2-HEK cells

Generation of HEK293 cells transfected to stably express CRTH2 tagged N-terminally with the FLAG-epitope tag (CRTH2-HEK) was described previously in detail [37]. Native HEK293 cells were obtained from the American Type Culture Collection (ATCC). CRTH2-HEK cells were cultivated in Dulbecco's modified Eagles medium (DMEM) supplemented with 10% (v/v) fetal bovine serum, 1% sodium pyruvate, 100 U/ml penicillin, 100 µg/ml streptomycin, and 400 µg/ml G418. Cells were kept at 37°C in a 5% CO_2_ atmosphere.

### Dynamic mass redistribution (DMR) assay in CRTH2-HEK293 cells

Dynamic mass redistribution assays were performed on a beta version of the Corning® Epic® Biosensor (Corning, NY, USA) as described previously in detail [34], [35]. The system detects changes in the local index of refraction upon stimulus-induced mass redistribution within the cell monolayer grown in 384-well Epic® microplates, which are equipped with a resonant wave guide grating biosensor at the bottom of each well. Cells were seeded at a density of 18,000 cells/well and cultivated for 20–24 h (37°C, 5% CO_2_) on fibronectin-coated Epic® biosensor plates. Cells were then washed twice with HBSS containing 20 mM HEPES and kept for 1 h in the Epic® reader at 28°C. The sensor plate was then scanned to obtain a base-line read prior to applying the compound solutions.

### Calcium ion mobilization assay in CRTH2-HEK293 cells

CRTH2-HEK cells were transiently transfected to co-express a chimeric Gαqi5 protein [38], [39] engineered to funnel signalling of Gi-sensitive receptors to the Gq signalling pathway using the calcium phosphate precipitation method as described previously [37]. 24 h after transfection cells were detached and replated into 96-well plates at a density of 80,000 cells per well. After 24 h cells were loaded with the Calcium 4 assay kit (Molecular Devices, CA, USA) and incubated for 30 min prior to challenge with PGH_1_, PGH_2_ or PGD_2_, respectively. Fluorescence output was measured in a NOVOstar® microplate reader with a built-in pipettor (BMG LabTech, Offenburg, Germany). Detection of fluorescence was initiated by injecting 20 µl of the respective agonist solution sequentially into separate wells.

### Human peripheral blood eosinophil purification

Blood was taken from healthy non-atopic volunteers according to a protocol approved by the Institutional Review Board of the Medical University of Graz and written informed consent was obtained from donors. Polymorphonuclear leukocytes (PMNL, including neutrophils and eosinophils) were prepared by dextran sedimentation of erythrocytes and by further centrifugation on Histopaque gradients. Eosinophils were purified from the PMNLs using negative magnetic selection with an antibody cocktail (CD2, CD14, CD16, CD19, CD56, and glycophorin A) and colloidal magnetic particles (StemCell Technologies, Vancouver, Canada) [40].

### Calcium ion mobilization assay in human eosinophils

Intracellular free Ca^2+^ levels in eosinophils were recorded by flow cytometry as described previously [41]. Polymorphonuclear leukocytes were incubated with 2 µM of the acetoxymethyl ester of the Ca^2+^ sensitive dye Fluo-3 and 0.02% pluronic F-127 for 60 minutes at room temperature. Cells were then stained with PE-conjugated anti-CD16 antibody in order to identify eosinophils as CD16-negative cells. Changes in intracellular Ca^2+^ levels were detected as the increase in fluorescence in the FL1-channel by flow cytometry.

### Internalization assay in CRTH2-HEK293 cells

CRTH2-HEK cells were grown to ∼80% confluence on glass coverslips pretreated with 1% gelatine. Cells were incubated with anti-FLAG® M1 antibody (1∶1,000) for 30 min at 37°C, then treated with either DMSO (0.1%) or 10 µM TM30089 for 15 minutes, followed by a 30 min exposure to 1 µM PGD_2_, 1 µM PGH_1_, or 10 µM TM30089. Cells were then rinsed once in PBS and fixed with 4% formaldehyde in phosphate-buffered saline. Following three washes in TBSC (137 mM NaCl, 25 mM Tris-base, 3 mM KCl, 1 mM CaCl_2_), the cells were permeabilized in blotto (3% milk, 0.1% Triton X-100, 50 mM Tris-HCl, pH 7.4), stained with Alexa Fluor 488-conjugated goat anti-mouse IgG_2b_ antibody (1∶500, 20 min), washed three times in TBSC, and mounted on glass microslides using Vectashield mounting medium. Confocal images were recorded using a Zeiss LSM 510 Meta laser scanning microscope.

### Eosinophil shape change assay

Eosinophil shape change was measured by an adaptation of a method originally developed by Sabroe and coworkers [42]. *Ethics Statement*. - The study was approved by National Health Service Oxfordshire Local Research Ethics Committee and written informed consent was obtained from donors. Heparinised blood was collected from healthy volunteers of unknown atopic status and incubated with red blood cell lysis buffer for 5 min at room temperature (300 µl of lysis buffer was used for every 100 µl of blood) followed by centrifugation at 300×*g* for 5 min. The supernatant was removed and leukocytes were resuspended in 50 ml PBS/2 mM EDTA. Cells were washed twice by centrifugation at 300×*g* for 5 min. Leukocytes were resuspended in RPMI/10% FCS. Fifty microliters of cells were added to a 96-well microtitre plate, containing 50 µl of test compounds. The plate was then incubated for 30 min at 37°C, 5% CO_2_. Subsequently, the plate was transferred to ice and the cell shape was fixed by addition of 150 µl cytofix buffer. Cell morphology was analysed using FACSArray. Eosinophils were gated based on their autofluorescence and 60,000 events were counted per sample.

### Culture of human pulmonary microvascular endothelial cells

Human pulmonary microvascular endothelial cells (HMVEC-L) cryopreserved in tertiary cultures were obtained from Lonza (Verviers, Belgium) and were maintained in EGM-2 MV Bullet kit medium (Lonza) supplemented with 5% FCS. 1% gelatine coating was applied to all culture surfaces to subserve endothelial cell attachment and growth. The medium was substituted every 2 days and cells were passaged upon 90% confluence (5–6 days); the cells were used maximally until passage 10 [43].

### Eosinophil adhesion to endothelial cells under flow conditions

Human pulmonary microvascular endothelial cells (4.3×10^5^/substrate) were grown on 1% gelatin-coated VenaEC biochips (Cellix, Dublin, Ireland). After reaching confluence the endothelial monolayers were superfused with 100 µl suspensions of 3×10^6^/ml eosinophils at 0.5 dyne/cm^2^ for 5 min at 37°C in the OKOLAB H201-T1 heated cage. Eosinophils were pretreated with 10 µM TM30089 (CRTH2 antagonist) or vehicle in endothelial medium for 10 min at room temperature followed by treatments with 1 µM PGH_1_, 30 nM PGD_2_ or vehicle for 10 min at 37°C prior the flow experiment. Cell adhesion was recorded by phase contrast on a Zeiss Axiovert 40 CFL microscope and a Zeiss A-Plan 10×/0.25 Ph1 lens, using Hamamatsu ORCA-03G digital camera and Cellix VenaFlux software. DucoCell analysis software (Cellix, Dublin) was applied for computerized image analysis where adherent eosinophils were quantified on every single image [40], [44].

### Culture of human CRTH2^+^CD4^+^ Th2 cells

Human CRTH2^+^CD4^+^Th2 cells were prepared using a modified method described previously [45]. The study was approved by National Health Service Oxfordshire Local Research Ethics Committee and written informed consent was obtained from donors. Briefly, peripheral blood mononuclear cells were collected from healthy blood donors with unknown atopic status (National Blood Service, Bristol, UK). Cells were isolated from buffy coats by Ficoll Hypaque (Amersham Biosciences) density gradient centrifugation, followed by CD4 cell purification using MACS CD4 T cell isolation kit II (Miltenyi Biotec, Surrey, UK). After 7 days of culture in X-VIVO 5 medium (Lonza, Basel, Switzerland) containing 10% human serum, 50 U/ml IL-2, and 100 ng/ml IL-4, CRTH2^+^ cells were isolated from the CD4 cultures by positive selection using an anti-human CRTH2 microbead kit. The harvested CD4^+^ CRTH2^+^ cells were treated as Th2 cells and were further amplified by stimulation with a T cell activation/expansion kit (Miltenyi Biotec, Surrey, UK) and grown in X-VIVO 15 medium containing 10% human serum and 50 U/ml IL-2 before use.

### Calcium ion mobilization assay in human Th2 cells

Human Th2 cells were washed once with Hank's Balanced Salt Solution (HBSS) and re-suspended in a FLIPR Calcium 5 loading buffer (Molecular Devices, CA, USA). The cells were aliquoted at 2×10^5^ cells/200 µl/well to a 96-well polylysine-coated black wall clear bottom plate and incubated for 60 min (37°C, 5% CO_2_) followed by incubation at room temperature for further 10 min and subsequently centrifuged at 600 rpm with brake off for 5 min. The changes in fluorescence after compound loading were measured by using a FlexStation (Molecular Devices, CA, USA) with run time 75 s at λ_ex_ = 485 nm and λ_em_ = 525 nm.

### Chemotaxis assays

For measurement of chemotaxis, Th2 cells were resuspended in X-VIVO 15 medium at 2×10^6^ cells/ml. The cell suspension (25 µl) and test samples (29 µl) prepared in X-VIVO 15 were applied to the upper and lower chambers of a 5-µm pore-sized 96-well ChemoTx plate (Neuro Probe, MD, USA). After 60 min incubation at 37°C, any cells remaining on top of the filter were wiped off and the plates were centrifuged at 300×*g* for 2 min to collect the cells on the underside of the filters. Cells were quantified by fluorescence activated cell sorting (FACS) with the FACSArray system (BD Biosiences, Oxford, UK). Background cell migration was determined by measuring the response to media alone.

### Calculations and Data Analysis

Calcium ion mobilization data in CRTH2-HEK cells are (i) solvent corrected and (ii) corrected for CRTH2-independent background responses as defined by the presence of the CRTH2-specific antagonist TM30089 (10 µM). All optical DMR recordings are (i) baseline corrected i.e., compound-induced wavelength shifts were corrected for signals obtained by addition of a compound-free solvent control and (ii) corrected for non-CRTH2-dependent background responses obtained upon compound addition into native HEK293 cells. Quantification of DMR signals was performed by calculation of the area under the curve (AUC) between 0 and 800 s. EC_50_ values were obtained by nonlinear regression analysis using Prism 4.02 (Graph Pad, San Diego, CA, USA). Statistical analysis was performed by two way analysis of variance (ANOVA) with Bonferroni's multiple comparison post-hoc testing using Prism® 4.02. P values were considered as significant (*) if p<0.05, as very significant (**) if p<0.01 and as extremely significant (***) if p<0.001.

## Results

**Figure 1:**
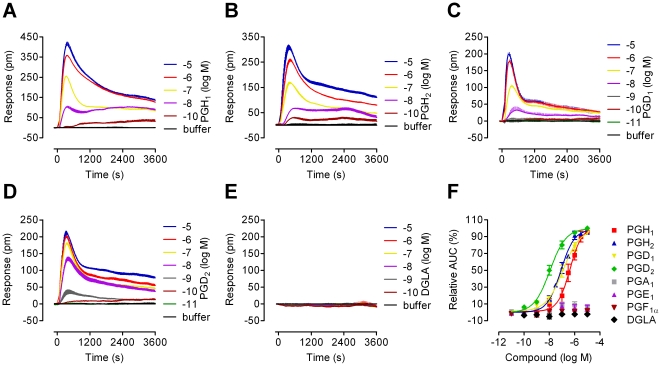
Prostaglandin H_1_ (PGH_1_) fully activates CRTH2 in living CRTH2-HEK cell transfectants. **A**–**E**, abilities of PGH_1_, selected prostaglandins, and the PGH_1_ precursor dihomo-γ-linolenic acid (DGLA) to stimulate CRTH2 signaling using dynamic mass redistribution (DMR) technology. Cells were challenged with increasing concentrations of the indicated ligands and DMR was recorded as a measure of receptor activity (representative optical traces). **F**, transformation of optical traces (**A**–**E**) into concentration effect curves. Molar log EC_50_ values were PGH_1_: −6.37±0.12; PGH_2_: −7.09±0.08; PGD_1_: −6.92±0.16; PGD_2_: −7.95±0.09 (mean values ± SEM, n = 3).

**Figure 2:**
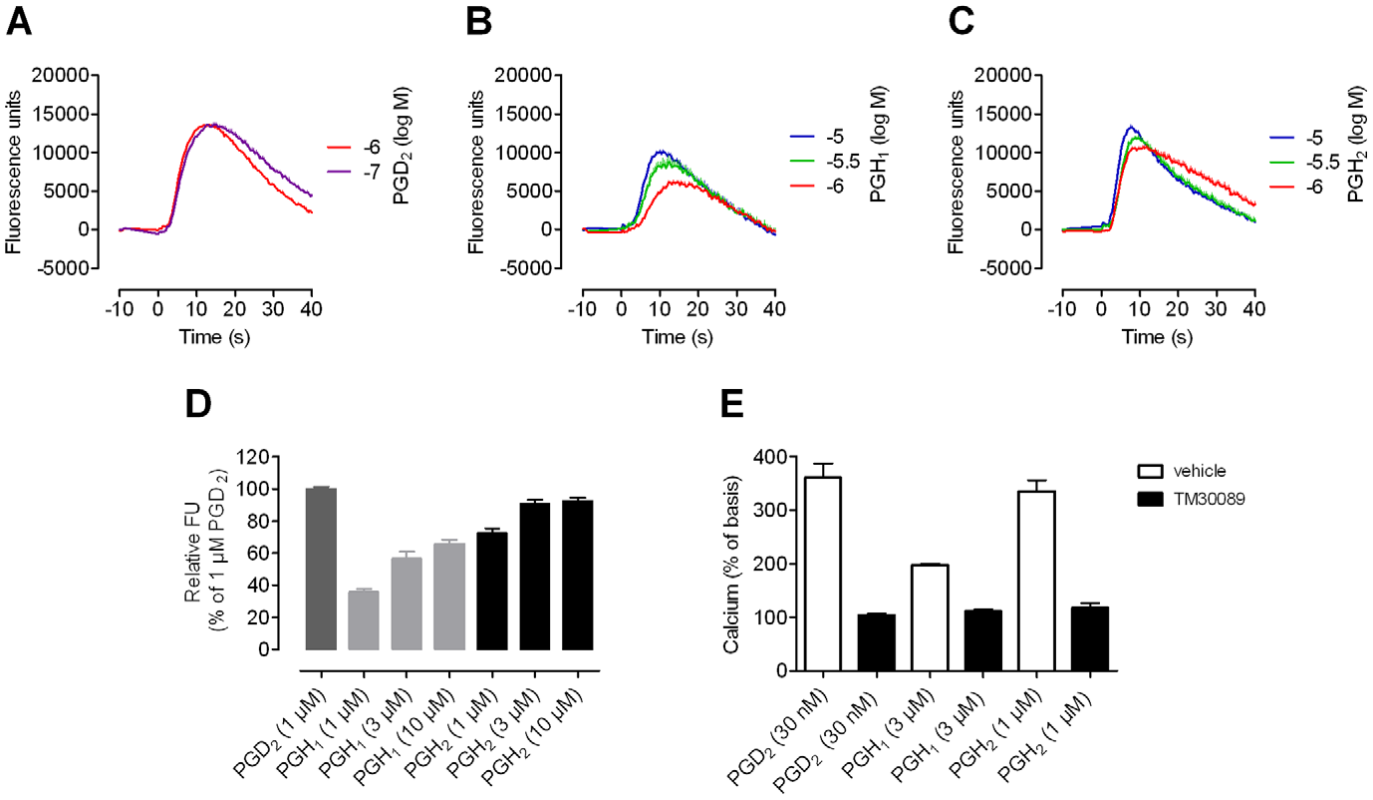
Prostaglandin H_1_ (PGH_1_) stimulates Ca^2+^ mobilization from intracellular stores in CRTH2 transfectants and primary human eosinophils. **A**–**D**: HEK293 cells stably expressing CRTH2 (CRTH2-HEK) were transiently transfected with a chimeric Gαqi5 protein to channel the Gi-sensitive CRTH2 receptor to mobilization of intracellular Ca^2+^. Cells were loaded with a Ca^2+^ fluorophore and CRTH2-specific Ca^2+^ traces were recorded over time upon challenge with the indicated agonists (**A**: PGD_2_, **B**: PGH_1_, **C**: PGH_2_). **A**–**C**: representative data (mean + SEM of triplicate determinations. **D**: Maximum responses of all experiments were normalized to Ca^2+^ flux induced by 1 µM PGD_2_ (mean + SEM, n = 3). **E**: PGH_1_ induces Ca^2+^ mobilization in human eosinophils via CRTH2. Intracellular free Ca^2+^ levels were quantified by flow cytometry as described in the methods section. The level of Ca^2+^ mobilization in response to vehicle without agonist was set to 100%. Ca^2+^ mobilization upon addition of PGH_1_, PGH_2_, and PGD_2_ is inhibited in the presence of 1 µM of the CRTH2 specific antagonist TM30089. Data are presented as the mean + SEM from 5 experiments conducted in triplicate, each experiment involving eosinophils from a separate donor.

To determine the ability of the DGLA-derived 1-series of prostaglandins (PGs) to stimulate CRTH2, HEK293 cells stably expressing CRTH2 (CRTH2-HEK cells) were treated with the various PGs and functional CRTH2 activity was monitored with an optical biosensor which captures receptor activation as dynamic mass redistribution (DMR) response [34], [35]. DMR assays allow for non-invasive, real-time recording of CRTH2 function immediately after ligand exposure. The known CRTH2 agonists PGD_2_ and PGH_2_ were included for comparison and reference purposes (chemical structures of all tested PGs are depicted in **Table S1**). PGH_1_ and PGD_1_, but not DGLA or any other 1-series PG, were capable of inducing CRTH2-mediated DMR with at least equal efficacy as PGD_2_ and PGH_2_ (**Figure 1A**–**E**). DMR was concentration-dependent and the potency of PGH_1_ was comparable to that of PGH_2_, although both PG precursors were less potent than PGD_2_ (**Figure 1F**). Importantly, PGH_1_ displayed no sign of decomposition during the DMR assay period (**Figure S2**). PGH_1_-activation of CRTH2 was also confirmed using Ca^2+^ mobilization assays in CRTH2-HEK cells (**Figure 2A**–**D**) and in primary human eosinophils that endogenously express CRTH2 (**Figure 2E**) [1]–[8]. These assays provided another measure of receptor activity within seconds after agonist exposure and therefore minimized the possibility of PGH_1_ modification during the assay period.

**Figure 3:**
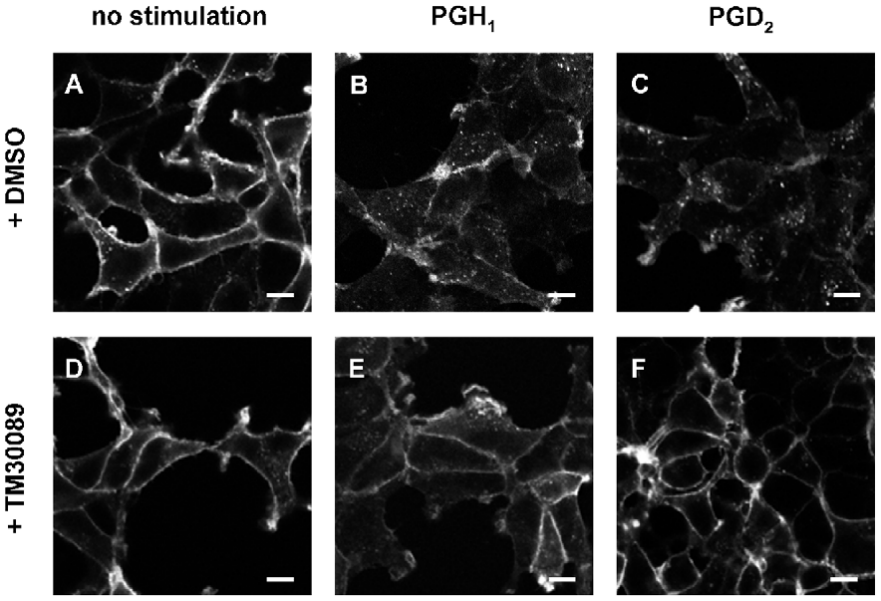
PGH_1_ promotes internalization of CRTH2. CRTH2-HEK cells were incubated with the M1 antibody recognizing the FLAG epitope tag fused in frame to the amino-terminus of CRTH2. Cells were then treated with either solvent control (**A**), 1 µM PGH_1_ (**B**) or 1 µM PGD_2_ (**C**) in the absence (**A**–**C**) or presence (**D**–**F**) of the CRTH2 antagonist TM30089 (10 µM). All ligand-stimulations were performed for 30 min at 37°C. Following stimulation, cells were fixed, permeabilized and immunostained with a fluorescent secondary antibody and imaged by confocal microscopy. Experiments were performed three times, and the shown images are representative of cell populations. Scale bars, 10 µm.

Most G protein coupled receptor (GPCR) agonists promote receptor internalization following activation [46], [47]. To corroborate our findings in yet another independent assay, PGH_1_ was tested for its ability to internalize CRTH2 using an ‘antibody feeding’ approach. Antibody feeding is a powerful technique that has been used elegantly to visualize receptor endocytosis and trafficking [48]–[50]. Living CRTH2-HEK293 transfectants engineered to express an extracellular FLAG-epitope tag were exposed to an anti-FLAG antibody under non-permeabilizing conditions such that only receptors at the surface membrane would be labelled during exposure to the antibody. Under these conditions, clear surface staining was observed for CRTH2-HEK cells but not for control HEK cells (not shown). To assess receptor internalization, CRTH2-HEK cells were first incubated with the anti-FLAG antibody, followed by stimulation with vehicle control (**Figure 3A**), PGH_1_ (**Figure 3B**) or PGD_2_ (**Figure 3C**) as a positive control. After fixation, the cells were permeabilized and the distribution of the anti-FLAG antibody-labeled receptors was detected with an Alexa-Fluor 488-labeled secondary antibody. Compared with vehicle-treated cells (**Figure 3A**), CRTH2 staining appeared at high density in intracellular vesicles upon PGH_1_ exposure, indicating that this ligand stimulated CRTH2 internalization, as did PGD_2_ (compare **Figure 3B and C**). The specificity of PGH_1_-CRTH2 interaction was further confirmed by using a selective CRTH2 antagonist (TM30089) [36], which abolished both PGH_1_ and PGD_2_-mediated CRTH2 internalization (**Figure 3E and F**) but was without effect when applied alone (**Figure 3D**).

**Figure 4:**
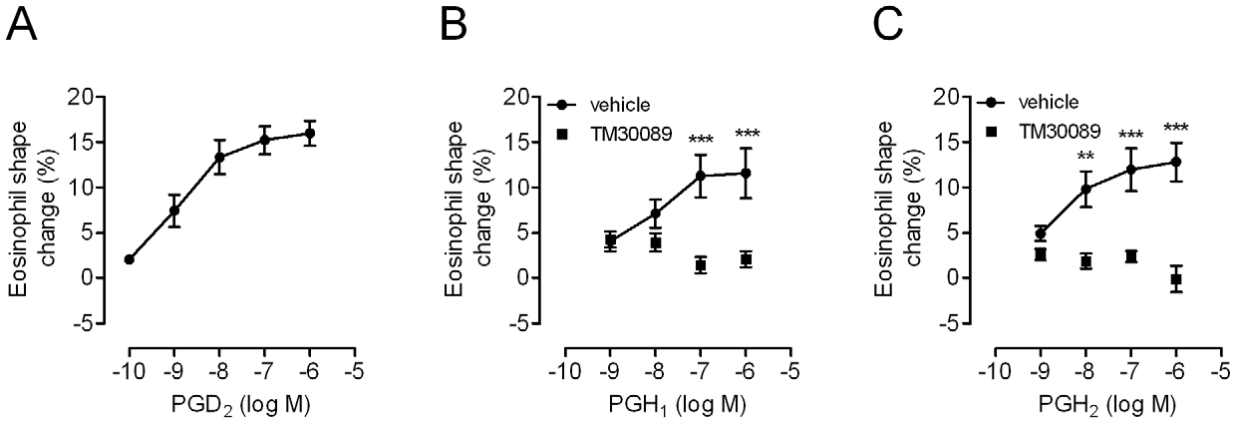
PGH_1_ activates human eosinophils via CRTH2. Human eosinophils were treated with the indicated concentrations of PGD_2_, PGH_1_, and PGH_2_, respectively, and chemotactic activation was measured in eosinophil shape change assays. Eosinophil shape change is inhibited in the presence of 1 µM of the CRTH2-specific antagonist TM30089. Note: rank order of PG potency matches well with the results obtained in CRTH2-HEK transfectants using DMR assays (compare with **Figure 1F**). Results are expressed as the mean ± SEM of 3 experiments conducted in triplicate with a separate donor used in each experiment. Statistical analysis was performed for vehicle vs. TM30089 treated cells and is indicated as (**) for p<0.01 and as (***) for p<0.001.

**Figure 5:**
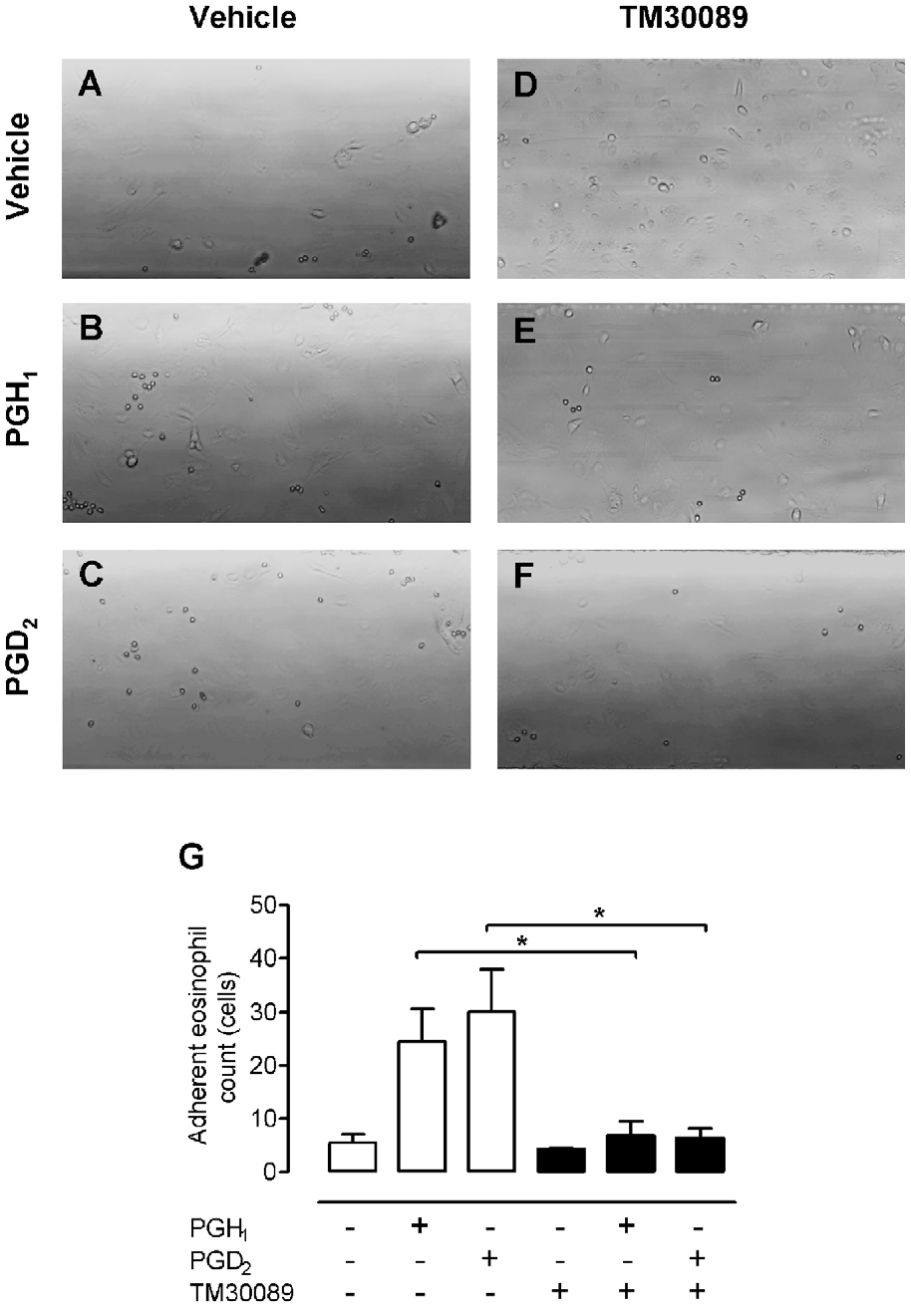
PGH_1_ induces eosinophil adhesion to human pulmonary microvascular endothelial cells under flow conditions. Eosinophils were pre-incubated with vehicle (**A**–**C**) or 10 µM CRTH2-specific antagonist TM30089 (**D**–**F**) for 10 min at room temperature followed by treatment with vehicle (**A**, **D**), 1 µM PGH_1_ (**B**, **E**) or 30 nM PGD_2_ (**C**, **F**) for 10 min at 37°C. Eosinophils were then superfused over human pulmonary microvascular endothelial cells grown on VenaEC biochips (Cellix, Dublin) for 5 min at 37°C. Representative images were taken 5 min after start of the superfusion (**A**–**F**). **G**: averaged data from **A**–**F**, quantified by computerized image analysis. Data are shown as mean + SEM of 4 experiments. *P<0.05 PGD_2_ versus TM30089+PGD_2_ and PGH_1_ versus TM30089+PGH_1_.

Given the predominant expression of CRTH2 in leukocytes such as eosinophils and Th2 cells, we investigated whether PGH_1_ acting through CRTH2 could mediate chemotactic activation of these cells. PGH_1_ was competent to induce shape change of human eosinophils and displays an efficacy comparable to that of PGH_2_, although both PGH derivatives demonstrated reduced efficacy compared to PGD_2_ (**Figure 4A**–**C**). The selective CRTH2 antagonist TM30089 inhibited the chemotactic activation of eosinophils, suggesting that this effect is mediated via CRTH2 (**Figure 4A**–**C**). Since activated eosinophils are thought to contribute to allergic inflammation by adhesion to the endothelium followed by extravasation, we examined whether PGH_1_ was able to modulate eosinophil-endothelial cell interaction under physiological flow conditions. To this end endothelial monolayers were grown to confluence on Cellix Vena EC biochip substrates for two days. Confluent endothelial monolayers were then superfused with purified eosinophils treated with vehicle, PGH_1_ or PGD_2_ as a reference and images were recorded for 5 min. PGH_1_ treatment of eosinophils significantly enhanced their adhesion to endothelial cells as did PGD_2_, and both agonists were ineffective in the presence of TM30089 (**Figure 5A**–**G**). Hence, PGH_1_ promotes eosinophil-endothelial cell adhesion via activation of CRTH2. Of note, PGH_1_-induced eosinophil adhesion to the endothelial layer is comparable to that induced by the chemokine eotaxin [40], suggesting that PGH_1_ does not represent an incomplete activator.

**Figure 6:**
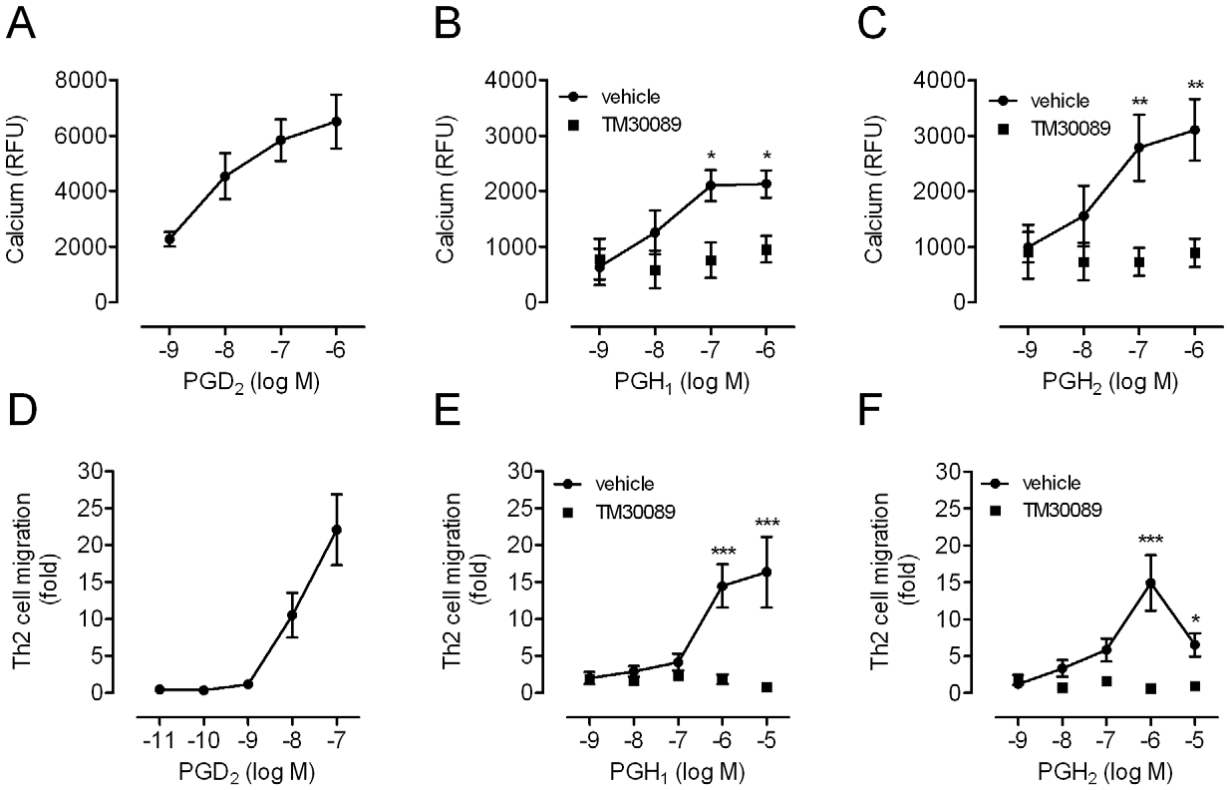
PGH_1_ activates human Th2 cells via CRTH2. Induction of Ca^2+^ mobilization (A–C) and cell migration (D–F) in human Th2 cells in response to the indicated concentrations of PGD_2_, PGH_1_, and PGH_2_, respectively. The level of cell migration in response to medium without agonist was set to 1 fold. Both Ca^2+^ mobilization and cell migration are inhibited in the presence of 1 µM of the CRTH2 specific antagonist TM30089. Pooled data is expressed as the mean ± SEM from 3 experiments conducted in duplicate, each experiment involving Th2 cells from a separate donor. Statistical analysis was performed for vehicle vs. TM30089 treated cells and is indicated as (*) for p<0.05, as (**) for p<0.01 and as (***) for p<0.001.

Agonist-induced elevation of intracellular Ca^2+^ is a key event for a variety of cellular processes in immune cells, and both PGD_2_ and the CRTH2 agonist indomethacin are known to be potent inducers of Ca^2+^ flux in Th2 cells [1], [51]. Whether H prostaglandins are also endowed with the ability to mobilize Ca^2+^ from intracellular stores in these cells has not been examined yet. Indeed, PGH_1_ and PGH_2_, as well as the reference agonist PGD_2_, induced robust and concentration-dependent Ca^2+^ responses in human Th2 cells in a CRTH2-dependent manner (**Figure 6A**–**C**). Ca^2+^ responses peaked at ∼50–60 s after compound addition (not shown) and provide further support for the notion that PGH_1_ itself but not a degradation product is responsible for the observed CRTH2 activation in human Th2 cells. In line with this notion, PGH_1_ was also competent to induce migration of Th2 cells in the absence but not in the presence of TM30089, as was also observed for PGH_2_ (**Figure 6D**–**F**). The hematopoietic PGD synthase inhibitor HQL79 did not prevent the pro-migratory effect of PGH_1_ ruling out that this response is due to conversion to PGD_1_ (**Figure S3**). As in the case of eosinophils, the efficacy of both PGH_1_ and PGH_2_ was lower than that of PGD_2_ in activating Th2 cells.

## Discussion

Prostaglandins can be produced from three different precursors, dihomo-γ-linolenic acid (DGLA), arachidonic acid (AA), and eicosapentaenoic acid (EPA), and the precursor determines which type of prostanoid is made, series 1, 2, or 3, respectively [52], [53]. The 2-series prostanoids are the biologically most active and abundant ones, and are commonly referred to as lipid mediators of inflammation [54], [55], [29]. In fact, there is substantial evidence to suggest that overproduction of AA-derived eicosanoids may play a detrimental role in a variety of inflammatory disorders, which is also reflected by the fact that COX-inhibitors are effective anti-inflammatory agents and the most common medication taken worldwide for the treatment of inflammation and inflammatory pain [56]–[58], [55], [59]–[61]. In contrast, eicosanoids derived from DGLA such as PGE_1_ or PGA_1_ are often viewed as ligands possessing anti-inflammatory properties [22], [62], [25], [27], and consequently, dietary supplementation of γ-linolenic acid to increase the level of DGLA - at the expense of AA-derived metabolites – has been proposed to improve clinical symptoms of inflammatory disorders [33]. So far, 1-series PGs have received rather limited attention which may relate, at least in part, to the inability of traditional ELISA methods to distinguish between series 1 and 2 ([32] and references therein). A very recent study, however, based on liquid chromatography-tandem mass spectrometry, identified prominent production of 1-series PGs in the supernatant of mouse peritoneal macrophages cultured in the presence of DGLA, a finding that is clearly suggestive of *in vivo* production.

Herein, we applied a non-invasive, label-free biosensor technology based on dynamic mass redistribution of cellular constituents to examine the “anti-inflammatory” 1-series of prostaglandins including their precursors DGLA and PGH_1_ for potential biological activity on the pro-inflammatory receptor CRTH2. We demonstrate for the first time that PGH_1_ and PGD_1_, but not PGA_1_, PGE_1_ or PGF_1α_, are functional agonists of CRTH2. We also provide evidence that CRTH2 activation by PGH_1_ can be detected in the receptor's native environment since PGH_1_ is capable of stimulating chemotactic activation of human eosinophils, and triggers their adhesion to endothelial cells under physiological flow conditions. PGH_1_ also positively regulates migration of human Th2 cells, and all of these effects are sensitive to inhibition by a CRTH2 specific antagonist.

Compared to PGD_2_, PGH_1_ acted as full agonist only in DMR assays on recombinant cells, but in all other functional readouts including those in primary eosinophils and Th2 cells, respectively, PGH_1_ behaved as a partial agonist. These data imply that PGH_1_ intrinsic activity may depend on both the assay system under study and the receptor density which is likely to be lower in cells expressing the receptor in its native environment. Nonetheless, these data clearly suggest that both eosinophils and Th2 cells can be activated by PGH_1_ in the absence of generation of endogenous PGD_2_. Whereas this study mainly focused on signal generation by the respective PGs, future studies may intend to discriminate PGH_1_ from other CRTH2 agonists by its ability to terminate signaling. PGH_1_, for example – unlike PGH_2_ – does not appear to be competent to switch off its own signaling at high agonist concentrations when migration of Th2 cells is the captured cellular event (compare **Figure 6E** with **F**). It will be interesting to unravel whether the closely related H PGs differ in their abilities to desensitize cellular responses by for example recruitment of β-arrestin proteins or activation of second messenger-dependent kinases.

CRTH2 is a remarkably promiscuous receptor, stimulated by PGD_2_ and several of its metabolites such as 13,14-dihydro-15-keto-PGD_2_, Δ^12^-PGD_2_, PGJ_2_, 15-deoxy-Δ^12,14^-PGJ_2_, and Δ^12^-PGJ_2_. All of these PGD_2_-derived ligands arise from the primary COX product PGH_2_ through isomerisation by two specific cytosolic enzymes: lipocalin-type PGD synthase (PGDS) and hematopoietic PGDS [63]–[65]. Accordingly, inhibition of PGDS is being considered as an opportune therapeutic strategy to treat inflammatory disorders [66]–[69]. More recently, however, additional lipid mediators generated from arachidonic acid independently of PGDS have been identified. Among those are PGF_2α_ [20], the thromboxane A_2_ metabolite 11-dehydro-thromboxane B_2_ [21] and PGH_2_, the precursor for all 2-series prostaglandins [70]. These data have strong implications for CRTH2 signaling in the absence of PGD_2_ production. Discovery of PGH_1_ as a functional agonist of CRTH2 is remarkable for two reasons: (i) because it is viewed as precursor for PGs with mainly anti-inflammatory properties such as PGA_1_ and PGE_1_ and (ii) because it is yet another lipid mediator triggering CRTH2 activation independent of PGDS. Whereas our data reinforce the potential importance of CRTH2 signaling in inflammatory diseases, they also raise the possibility that efficient suppression of inflammation may require pharmacological inhibition of CRTH2 rather than abrogation of PGDS activity.

As CRTH2 is activated by the 2-series PGs containing D, J, and F-rings, but not A or E-rings [17], it is of interest to determine whether the same structural requirements are also valid for the 1-series of PGs. Interestingly, only PGD_1_ - but not PGA_1_, PGE_1_, and PGF_1α_ - were found to activate CRTH2. These data suggest that CRTH2 bioactivity is not exclusively governed by the ring systems, but also by the number of double bonds, since PGF_2α_ and PGF_1α_ share identical rings, but differ only in saturation of their carbon chain.

Anti-inflammatory effects of 1-series PGs, in particular PGA_1_ and PGE_1_ have been repeatedly demonstrated *in vivo* and *in vitro* in diverse cell types and animal models [22], [23], [27], [71]. PGA_1_, for example, has been shown to limit inflammatory responses in activated monocytes/macrophages via induction of anti-inflammatory cytokine IL-10 expression [71] and to suppress NFkappaB activation which in turn is essential for COX-2 gene expression [27]. PGE_1_, on the other hand, appears to possess anti-inflammatory properties which differ from arachidonic-acid-derived PGE_2_ and which favourably affect a variety of inflammatory conditions: PGE_1_ alleviates inflammation in rat adjuvant arthritis model [22] and in a mouse lupus model [24], suppresses immune complex vasculitis [23], inhibits collagenase activity [62] and ameliorates inflammatory skin diseases [72]. In this context it is noteworthy that PGH_1_, the precursor for these two anti-inflammatory PGs, is a potent and efficacious activator of the pro-inflammatory receptor CRTH2. Generation of PGH_1_ requires cyclo-oxygenases, but not PG synthases, suggesting that generation of this CRTH2 ligand is not restricted to cells that also co-express PGD synthases. Although the exact physiological concentrations of H prostaglandins in the extracellular space at the site of inflammation may be difficult to determine, there is evidence that H prostaglandins do not only serve as intracellular substrates for PG synthases, but may also be secreted from cells in an untransformed manner [73]–[78]. Hence, the results presented herein not only have identified PGH_1_ as novel CRTH2 ligand; they also at least in part provide a proof of principle that PGH_1_ may be competent to promote allergic inflammation via stimulation of CRTH2. Our findings may be particularly relevant when fatty acid composition of cell membrane phospholipids, and hence potential eicosanoid formation, are intended to be altered with diets enriched in γ-linolenic acid or DGLA to foster production of putative anti-inflammatory eicosanoids. Indeed, DGLA content in serum phospholipids has been found to negatively influence lung function parameters in asthmatic subjects [79]. A positive association between DGLA plasma levels and the occurrence of asthma was also found in young adults [80]. Although the precise mechanisms underlying these clinical observations have not been elucidated, it is tempting to speculate that CRTH2-activation by PGH_1_ may also contribute to the clinical phenotype.

Taken together, our results identify PGH_1_ as potent and efficacious activator of the pro-inflammatory receptor CRTH2 and strengthen the role of this receptor as an important player in allergic inflammation, and hence attractive therapeutic target for the treatment thereof.

## Supporting Information

Figure S1 **Pathways of eicosanoid production and their interaction with cellular effector proteins.** Dihomo-γ-linolenic acid (DGLA) and arachidonic acid (AA) are converted to the indicated prostaglandins (PG) and thromboxanes (TX). Cyclo-oxygenase enzymes 1 and 2 (COX-1/2) convert the precursors DGLA and AA to PGH_1_ and PGH_2_, respectively, which are acted upon by specific prostaglandin and thromboxane synthases to either yield the 1-series or the 2-series of eicosanoids. 2-series PGs known to display relevant affinity to CRTH2 are indicated in green. 1-series PGs with relevant activity on CRTH2 are indicated in blue.
(TIF)

Figure S2 **Biosensor fingerprints indicate that PGH_1_ does not decompose during the real-time functional DMR assay.** Given the reported instability of PGH_1_, we examined the possibility that the ligand might degrade during the assay period giving rise to its isomerization products PGE_1_ (inactive at CRTH2) and PGD_1_ (active at CRTH2). To this end, optical CRTH2 traces were recorded in CRTH2-HEK cells after adding PGH_1_ which was (**A**) freshly prepared or (**B**) pre-incubated in an aqueous solution on CRTH2-HEK cells at a temperature of 28°C for 60 min: DMR traces (**A** and **B**) are virtually superimposable. (**C**) Decomposition of PGH_1_ can be excluded since PGD_1_ induces optical traces distinct in shape from those triggered by PGH_1_, and also, PGE_1_ does not display any bioactivity on CRTH2 (see **Figure 1F**); hence PGH_1_ is not biotransformed by the cells during the course of the DMR experiments. PGH_1_ was applied at 3 µM and PGD_1_ at 1 µM final concentration. Shown are representative traces + SEM of at least three independent experiments performed in triplicates.
(TIF)

Figure S3 **Inhibition of PGD synthase does not alter the ability of PGH_1_ to mediate chemotaxis of Th2 cells.** Migration of human Th2 cells in response to various concentrations of PGH_1_ in the absence or presence of 10 µM HQL-79 was measured as described in the methods section. Data are expressed as mean ± SEM of 3 independent experiments. The level of cell migration in response to medium without PGH_1_ in each experiment was set to 1 fold.
(TIF)

Table S1 **Chemical structures of eicosanoids and the CRTH2 antagonist TM30089.**
(TIF)
