# Supplementary figures and images for: PGH1, the Precursor for the Anti-Inflammatory Prostaglandins of the 1-series, Is a Potent Activator of the Pro-Inflammatory Receptor CRTH2/DP2
Source: PLoS One. 2012 Mar 19;7(3):e33329. doi: 10.1371/journal.pone.0033329 (PMC3307725; doi:10.1371/journal.pone.0033329)

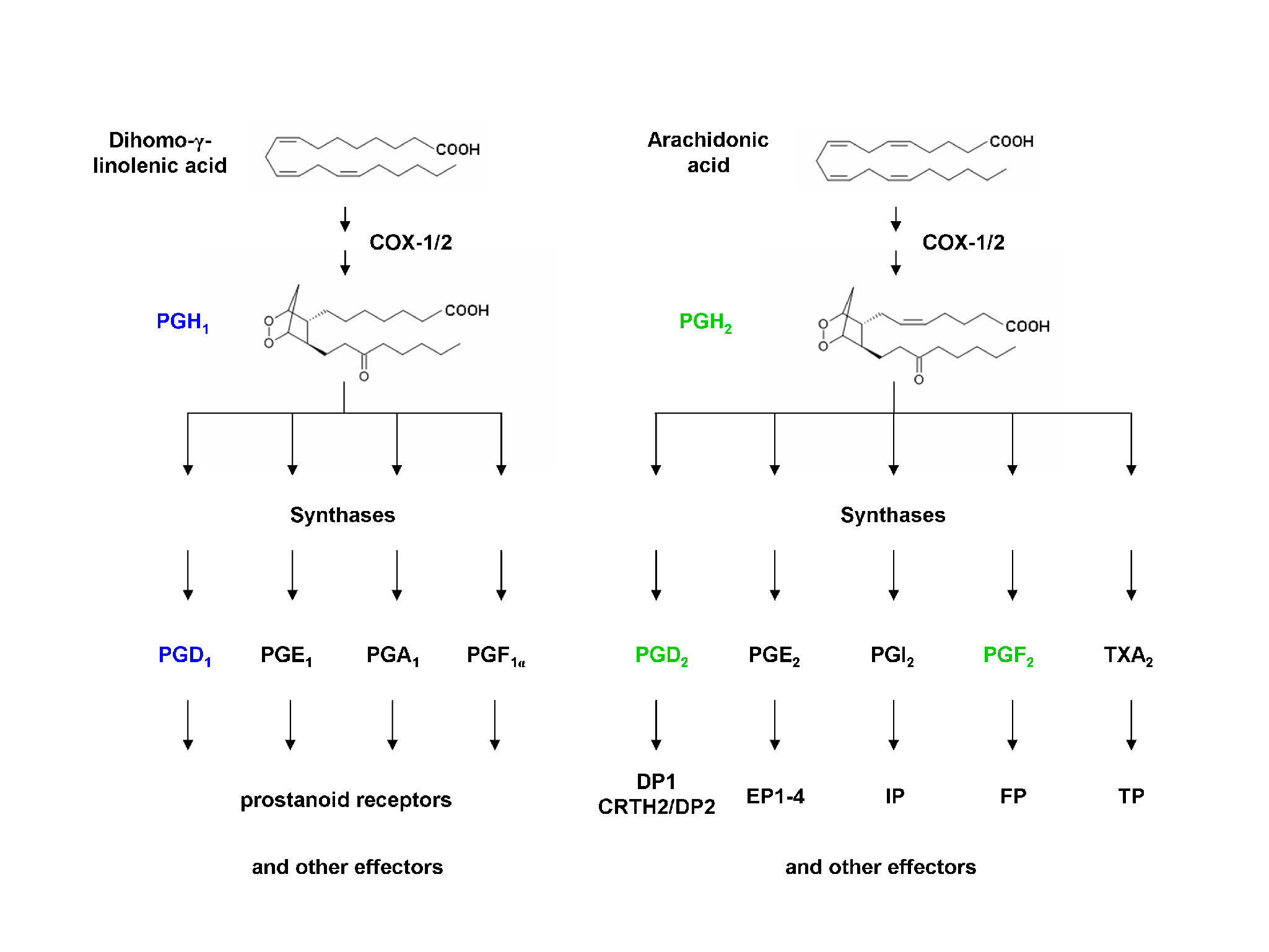

Supplement: Figure S1 — Pathways of eicosanoid production and their interaction with cellular effector proteins. Dihomo-γ-linolenic acid (DGLA) and arachidonic acid (AA) are converted to the indicated prostaglandins (PG) and thromboxanes (TX). Cyclo-oxygenase enzymes 1 and 2 (COX-1/2) convert the precursors DGLA and AA to PGH1 and PGH2, respectively, which are acted upon by specific prostaglandin and thromboxane synthases to either yield the 1-series or the 2-series of eicosanoids. 2-series PGs known to display relevant affinity to CRTH2 are indicated in green. 1-series PGs with relevant activity on CRTH2 are indicated in blue. (TIF) [file pone.0033329.s001.tif]

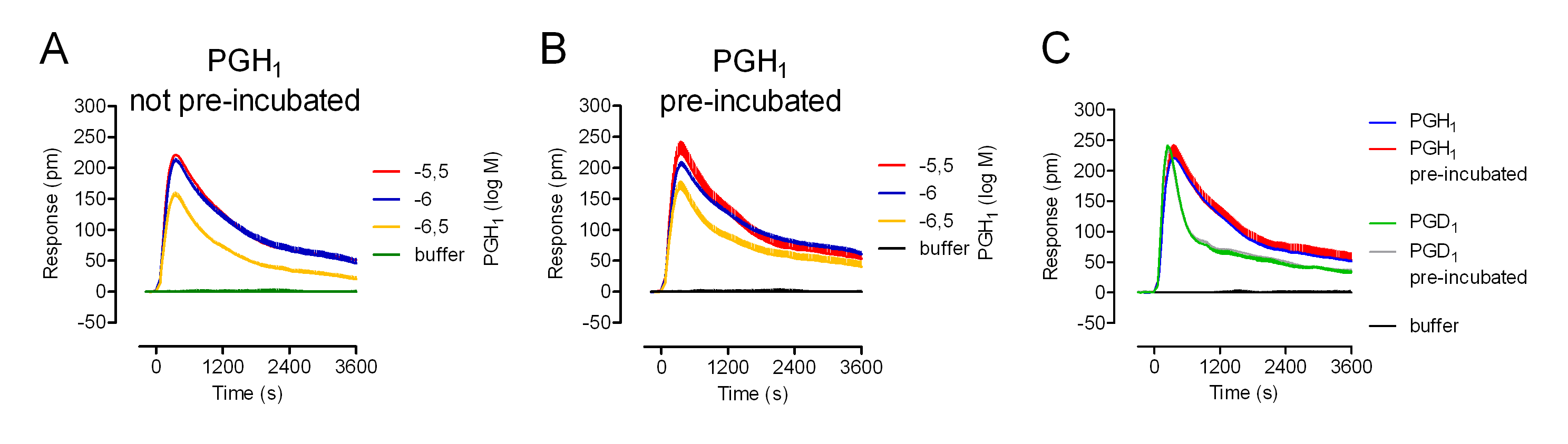

Supplement: Figure S2 — Biosensor fingerprints indicate that PGH1 does not decompose during the real-time functional DMR assay. Given the reported instability of PGH1, we examined the possibility that the ligand might degrade during the assay period giving rise to its isomerization products PGE1 (inactive at CRTH2) and PGD1 (active at CRTH2). To this end, optical CRTH2 traces were recorded in CRTH2-HEK cells after adding PGH1 which was (A) freshly prepared or (B) pre-incubated in an aqueous solution on CRTH2-HEK cells at a temperature of 28°C for 60 min: DMR traces (A and B) are virtually superimposable. (C) Decomposition of PGH1 can be excluded since PGD1 induces optical traces distinct in shape from those triggered by PGH1, and also, PGE1 does not display any bioactivity on CRTH2 (see Figure 1F ); hence PGH1 is not biotransformed by the cells during the course of the DMR experiments. PGH1 was applied at 3 µM and PGD1 at 1 µM final concentration. Shown are representative traces + SEM of at least three independent experiments performed in triplicates. (TIF) [file pone.0033329.s002.tif]

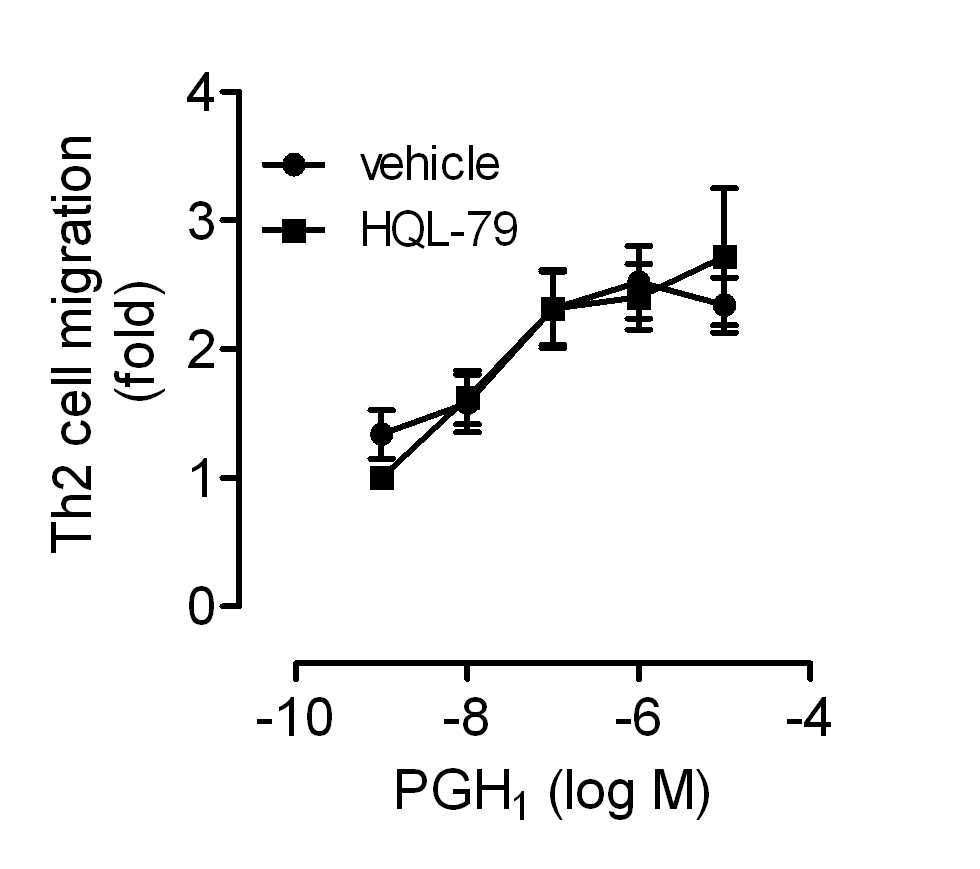

Supplement: Figure S3 — Inhibition of PGD synthase does not alter the ability of PGH1 to mediate chemotaxis of Th2 cells. Migration of human Th2 cells in response to various concentrations of PGH1 in the absence or presence of 10 µM HQL-79 was measured as described in the methods section. Data are expressed as mean ± SEM of 3 independent experiments. The level of cell migration in response to medium without PGH1 in each experiment was set to 1 fold. (TIF) [file pone.0033329.s003.tif]

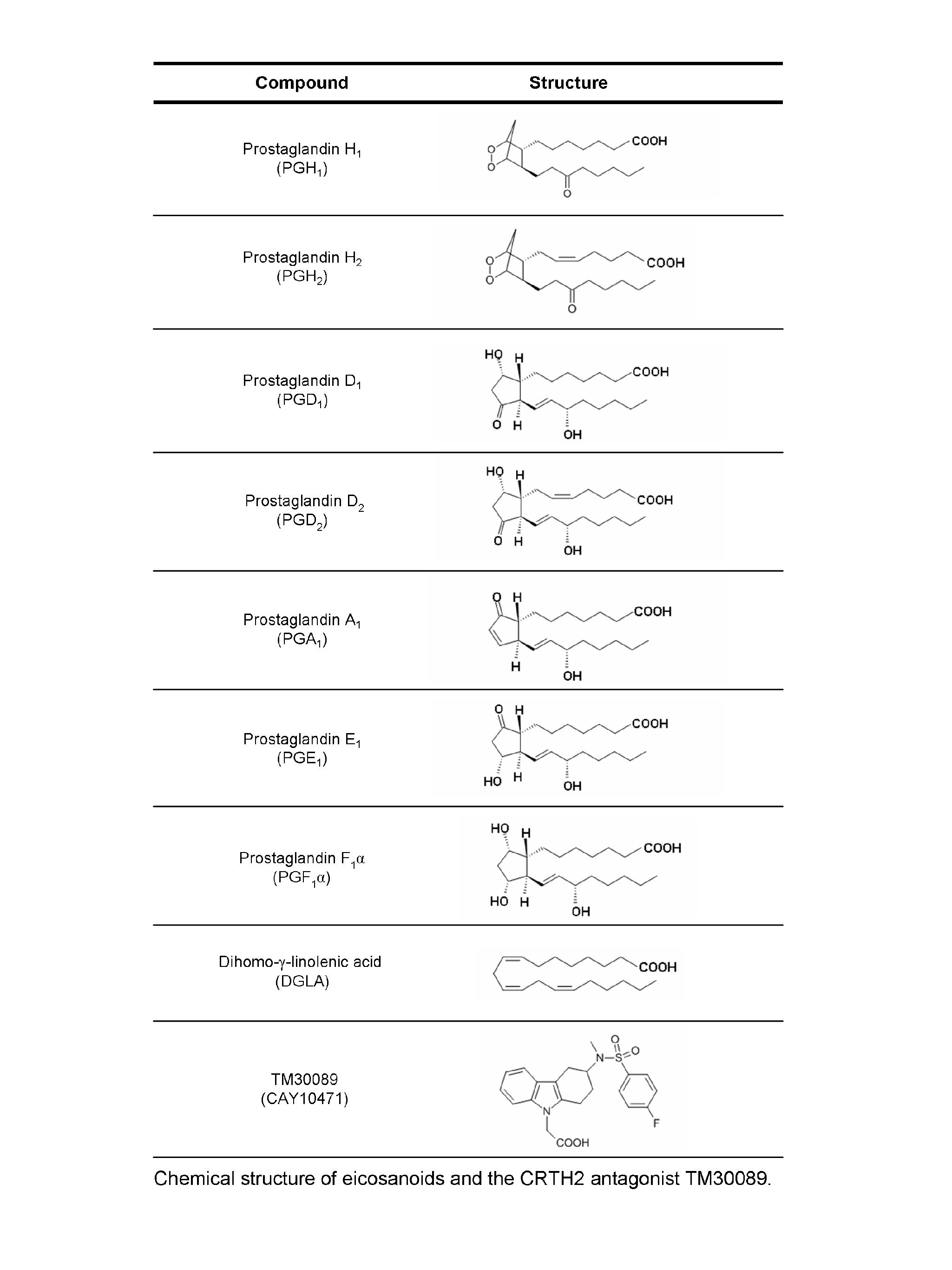

Supplement: Table S1 — Chemical structures of eicosanoids and the CRTH2 antagonist TM30089. (TIF) [file pone.0033329.s004.tif]
